# Impact of HIV on CD8+ T Cell CD57 Expression Is Distinct from That of CMV and Aging

**DOI:** 10.1371/journal.pone.0089444

**Published:** 2014-02-27

**Authors:** Sulggi A. Lee, Elizabeth Sinclair, Hiroyu Hatano, Priscilla Y. Hsue, Lorrie Epling, Frederick M. Hecht, David R. Bangsberg, Jeffrey N. Martin, Joseph M. McCune, Steven G. Deeks, Peter W. Hunt

**Affiliations:** 1 Departments of Medicine, Epidemiology and Biostatistics, University of California San Francisco, San Francisco, California, United States of America; 2 Department of Medicine, Massachusetts General Hospital and Harvard School of Public Health, Harvard Medical School, Boston, Massachusetts, United States of America; 3 Department of Medicine, Mbarara University of Science and Technology, Mbarara, Uganda; University of Cape Town, South Africa

## Abstract

**Background:**

Chronic antigenic stimulation by cytomegalovirus (CMV) is thought to increase “immunosenesence” of aging, characterized by accumulation of terminally differentiated CD28- CD8+ T cells and increased CD57, a marker of proliferative history. Whether chronic HIV infection causes similar effects is currently unclear.

**Methods:**

We compared markers of CD8+ T cell differentiation (e.g., CD28, CD27, CCR7, CD45RA) and CD57 expression on CD28- CD8+ T cells in healthy HIV-uninfected adults with and without CMV infection and in both untreated and antiretroviral therapy (ART)-suppressed HIV-infected adults with asymptomatic CMV infection.

**Results:**

Compared to HIV-uninfected adults without CMV (n = 12), those with asymptomatic CMV infection (n = 31) had a higher proportion of CD28-CD8+ T cells expressing CD57 (P = 0.005). Older age was also associated with greater proportions of CD28-CD8+ T cells expressing CD57 (rho: 0.47, P = 0.007). In contrast, untreated HIV-infected CMV+ participants (n = 55) had much lower proportions of CD28- CD8+ cells expressing CD57 than HIV-uninfected CMV+ participants (P<0.0001) and were enriched for less well-differentiated CD28- transitional memory (T_TR_) CD8+ T cells (P<0.0001). Chronically HIV-infected adults maintaining ART-mediated viral suppression (n = 96) had higher proportions of CD28-CD8+ T cells expressing CD57 than untreated patients (P<0.0001), but continued to have significantly lower levels than HIV-uninfected controls (P = 0.001). Among 45 HIV-infected individuals initiating their first ART regimen, the proportion of CD28-CD8+ T cells expressing CD57 declined (P<0.0001), which correlated with a decline in percent of transitional memory CD8+ T cells, and appeared to be largely explained by a decline in CD28-CD57- CD8+ T cell counts rather than an expansion of CD28-CD57+ CD8+ T cell counts.

**Conclusions:**

Unlike CMV and aging, which are associated with terminal differentiation and proliferation of effector memory CD8+ T cells, HIV inhibits this process, expanding less well-differentiated CD28- CD8+ T cells and decreasing the proportion of CD28- CD8+ T cells that express CD57.

## Introduction

Despite effective antiretroviral therapy (ART), HIV-infected individuals remain at higher risk for aging-related diseases (e.g., heart disease, cancer, and bone disease) and death than the general population [1]. HIV also causes several defects in the immune system that appear similar to those observed in elderly populations, which has raised the hypothesis that HIV causes accelerated aging of the immune system, or “immunosenescence [1].” T cell senescence, whether driven by aging and/or by chronic antigenic stimulation from pathogens such as cytomegalovirus (CMV), is typically characterized by the accumulation of terminally differentiated CD8+ T cells with shortened telomeres, the loss of expression of the co-stimulatory molecule CD28, and increased expression of CD57, a marker of proliferative history and poor proliferative capacity [2].

While the loss of CD28 expression on CD8+ T cells is characteristic of HIV infection, the impact of HIV on CD57 expression on CD8+ T cell subsets – particularly the effector memory CD8+ T cell subsets that normally express CD57 - is less well established. HIV-specific CD8+ T cells are more likely to express CD57 than non-HIV-specific CD8+ T cells [3], and CD57 expression is increased on the total memory CD8+ T cell population in HIV infection [4], [5], but much of this increase could be explained by relative enrichment for effector CD28- CD8+ T cells over central memory and naïve CD8+ T cells (which rarely express CD57). Indeed, a recent report suggests that an abnormally low proportion of terminally differentiated CD45RA+ CD28-CCR7- CD8+ T cells express CD57 in untreated HIV infection, consistent with the possibility that these cells had completed fewer prior rounds of proliferation *in vivo* [6]. CD57 expression was also a much less consistent correlate of clinical progression in untreated HIV infection than the loss of CD28 expression on CD8+ T cells [4].

In the current study, we sought to better characterize the effect of HIV on the proportion of CD28-CD8+ T cells expressing CD57 and to compare these effects with those observed in aging and chronic asymptomatic CMV infection. Our data suggest that asymptomatic CMV infection and advancing age are both associated with increased terminal differentiation and a high proportion of CD28-CD8+ T cells expressing CD57, consistent with the “immunosenescent” phenotype of aging. In contrast, untreated HIV infection leads to enrichment for less well-differentiated transitional cells and lower proportions of CD28-CD8+ T cells expressing CD57. Though these abnormalities improve during suppressive ART, they fail to normalize in chronically HIV-infected individuals. Collectively, these data suggest that the phenotypic CD8+ T cell abnormalities of untreated and treated HIV infection are distinct from those of chronic CMV infection and aging.

## Materials and Methods

### Ethics statement

All participants provided written informed consent, this research was approved by the institutional review boards of the University of California, San Francisco and the Mbarara University of Science and Technology, and all studies were conducted according to the principles expressed in the Declaration of Helsinki.

### Participants

For the cross-sectional comparisons, HIV-uninfected participants with and without asymptomatic CMV infection as well as chronically untreated HIV-infected patients (>1 year following sero-conversion) were sampled from the SCOPE and OPTIONS cohorts in San Francisco as previously described [7], [8]. HIV-infected individuals maintaining ART-mediated viral suppression (<75 copies HIV RNA/ml plasma) were sampled from the SCOPE cohort. For longitudinal assessment of changes in T cell subsets during early ART-mediated viral suppression, participants were also sampled from the Uganda Antiretroviral Treatment Outcomes (UARTO) cohort, a rural, clinic-based cohort of 750 chronically HIV-infected patients initiating their first ART regimen at the Mbarara University of Science and Technology HIV Clinic, Uganda.

### Laboratory methods

T cell phenotyping was performed on cryopreserved peripheral blood mononuclear cells (PBMCs), as described previously [9]. Cells were thawed, washed, stained with LIVE/DEAD® Fixable Aqua Dead Cell Stain Kit (Invitrogen), excluding non-viable cells and stained with fluorescently-conjugated monoclonal antibodies including: CD3-Pacific Blue and CD28-PE-Cy™5 (BD Pharmingen); CD8-QDOT®605 and CD4-PE-Texas Red® (Invitrogen); CD45RA-PE, CD31-FITC, CCR7-PE-CY™7 (all BD Bioscences); CD57-Alexa Fluor ®647 (Biolegend); and CD27-APCeFluor®780 (eBioscience). Cells were fixed in 0.5% formaldehyde and data were acquired on a LSR II Flow cytometer (BD Biosciences), with ≥250,000 lymphocytes collected. CPT beads (BD Bioscience) were used for instrument set up for each run and Rainbow beads (Spherotec) for standardized instrument settings between runs. Aliquots of a control specimen were thawed with every run and used to confirm run-to-run reproducibility. FMO controls were also prepared on controls for each run to check that gates were set consistently between runs. Data was compensated and analyzed in FlowJo V9 (TreeStar). Gates were set to define positive expression of each maturation marker and Boolean gates were calculated to determine the %CD8+ T cells that expressed combinations of CD45RA, CCR7, CD27, CD28 and CD57. For some populations, results obtained from Boolean combinations were confirmed by gating directly visualized populations to confirm the validity of the Boolean gating approach. Maturational T cell subsets were defined as naïve T_N_ (CD28+CD27+CCR7+CD45RA+), central memory T_CM_ (CD28+CD27+CCR7+CD45RA-), as well as the following CD28- populations: transitional T_TR_ (CD28-CD27+CCR7-CD45RA-), effector memory, T_EM_ (CD28-CD27-CCR7-CD45RA-), and terminally differentiated, T_EMRA_ (CD28-CD27-CCR7-CD45RA+). Chronic asymptomatic CMV infection was confirmed as a positive CMV IgG titer and for a subset of HIV-infected participants without available CMV serology, >0.1% pp65/IE-specific IFN-γ+ CD8+ T cell responses by cytokine flow cytometry (10-fold increase over limit of detection) as previously described [10].

### Statistical methods

Cross-sectional pairwise comparisons between groups were performed using Wilcoxon rank sum tests. Changes in T cell subsets during the first six months of ART-mediated viral suppression in the UARTO cohort were analyzed using Wilcoxon signed rank tests. Adjusted comparisons were assessed with linear regression. Since age is an important determinant of CD57 expression, we considered age a confounder in all adjusted analyses. Since some comparator groups had insufficient female participants to assess gender effects, we restricted adjusted analyses to male participants. Since CMV is an important determinant of CD57 expression, and since almost all HIV-infected participants were CMV co-infected, we restricted all comparisons between HIV+ and HIV- participants to those with confirmed asymptomatic CMV infection. Relationships between continuous variables were assessed with Spearman's rank order correlation coefficients. Variables in logistic and/or linear regression models were transformed as necessary to satisfy model assumptions. Statistical analyses were performed using STATA Version 12 (StataCorp LP, Texas), 2011.

## Results

### Impact of CMV and age on CD57 expression in HIV-uninfected Individuals

**Figure 1:**
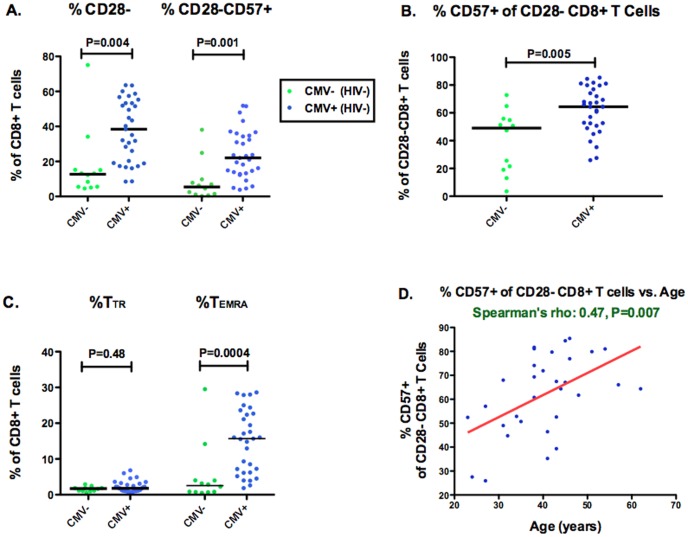
Effects of CMV and age on the proportion of CD28-CD8+ T cells expressing CD57. The proportion of CD28- and CD28-CD57+ CD8+ T cells (**A**), of CD28-CD8+ T cells expressing CD57 (**B**), and of maturational subsets of CD28- CD8+ T cells: transitional memory, T_TR_, (CD28-CD27+CCR7-CD45RA-) and terminally differentiated, T_TEMRA_, (CD28-CD27-CCR7-CD45RA+) CD8+ T cells (**C**), were compared between HIV-uninfected asymptomatic CMV-seronegative (green) and CMV-seropositive (blue) participants. Bars represent median values. The correlation between the proportion of CD28-CD8+ T cells expressing CD57 and age was also assessed among CMV-seropositive HIV-uninfected adults (**D**). The red line represents a linear prediction.

We first assessed the impact of CMV and age on CD8+ T cell CD28 and CD57 expression in 43 healthy, asymptomatic HIV-uninfected participants with a median age of 44 (Table 1). Since nearly all CD57+ CD8+ T cells are CD28- (Figure S1), we assessed not only the proportion of CD28-CD57+ CD8+ T cells (a commonly reported senescence phenotype in the aging literature), but also the proportion of CD28-CD8+ T cells that express CD57. Compared to CMV-seronegative participants (n = 12), CMV-seropositive participants (n = 31) had a higher median percent CD28- CD8+ T cells (38% vs. 13%, P = 0.0004), percent CD28-CD57+ CD8+ T cells (22% vs. 5%, P = 0.001), and proportion of CD28-CD8+ T cells expressing CD57 (64% vs. 49%, P  =  0.005, Figure 1A–B). Much of this enrichment appeared to be explained by an expansion of CD28-CD57+ CD8+ T cell counts in CMV-seropositive compared to CMV-seronegative participants (median 63 vs. 24 cells/mm^3^, P = 0.01, data not shown). All of these differences remained significant after adjustment for age and restricting to men (P≤0.009 for all). Asymptomatic CMV infection was also associated with enrichment for terminally differentiated, T_EMRA_, (CD28-CD27-CCR7-CD45RA+) CD8+ T cells (P = 0.0004, Figure 1C), which also remained significant after adjustment for age and restricting to men (P = 0.001).

**Table 1:** Characteristics of SCOPE and OPTIONS participants by CMV and HIV status.

SCOPE and OPTIONS	HIV-CMV-Median (IQR) (n = 12)	HIV-CMV+ Median (IQR) (n = 31)	*HIV+ART+ Median (IQR) (n = 96)	*HIV+ART-Median (IQR) (n = 55)
**Age, years** **	35 (34–50)	41 (34–45)	51 (45–57)	40 (34–44)
**Male gender, No. (%)**	11 (92%)	29 (94%)	84 (88%)	52 (95%)
**Proximal CD4+ count, cells/mm^3^** **	NA	NA	592 (448–738)	407 (314–504)
**Proximal HIV RNA level, log_10_copies/mL** **	NA	NA	≤1.6	4.4 (4.1–4.6)
**Pre-ART nadir CD4+ count, cells/mm^3^**	NA	NA	110 (49–180)	NA
**Duration of viral suppression, years**	NA	NA	4 (2–9)	NA

^*^ All CMV+.

^**^ Difference among groups (Kruskal-Wallis or Wilcoxon ranksum test), P<0.001.

Increasing age was also associated with these T cell abnormalities, even in this relatively young sample of HIV-uninfected individuals. Older age was strongly associated with a higher proportion of CD28-CD8+ T cells expressing CD57 in the subset of CMV-seropositive HIV-uninfected participants (rho: 0.47, P = 0.007, Figure 1D), but there was no evidence for an association between age and the %CD28- CD8+ T cells (rho: 0.01, P = 0.97) or the %T_EMRA_ CD8+ T cells (rho: 0.08, P = 0.67). Among all HIV-uninfected participants, each ten-year increase in age was associated with a mean 2% increase in the proportion of CD28-CD8+ T cells expressing CD57(P = 0.04), after adjusting for CMV status. We lacked sufficient numbers of CMV seronegative individuals to assess whether CMV serostatus modifies the association between age and the proportion of CD28-CD8+ T cells expressing CD57.These data confirm that CMV and age increase the proportion of CD28- CD8+ T cells that express CD57 and extend previous findings in elderly cohorts [11] to a cohort of much younger adults.

### Impact of chronic HIV infection and ART on CD57 expression

**Figure 2:**
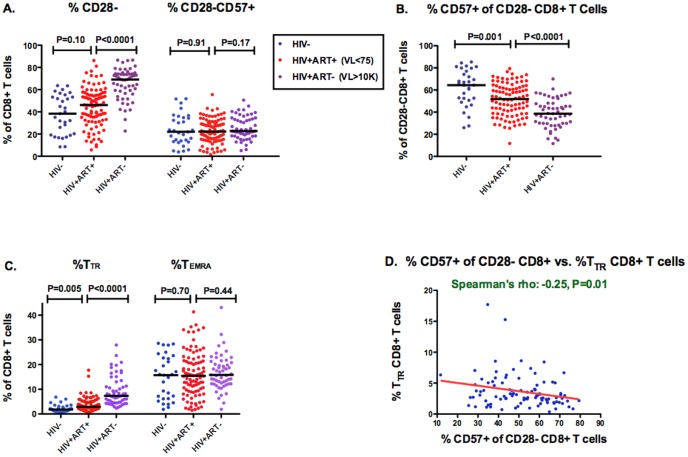
Effects of HIV and ART on CD8+ T cell subset counts. Total CD8+ T cell and CD28- CD8+ T cell (**A**), CD28-CD57+ and CD28-CD57-CD8+ T cell (**B**), and transitional memory, T_TR_, (CD28-CD27+CCR7-CD45RA-) and terminally differentiated, T_TEMRA_, (CD28-CD27-CCR7-CD45RA+) CD8+ T cell (**C**) counts were compared between HIV-uninfected (blue), ART-suppressed (red), and untreated viremic (purple) HIV-infected individuals. All comparisons were restricted to CMV-infected participants. Bars represent median values.

**Figure 3:**
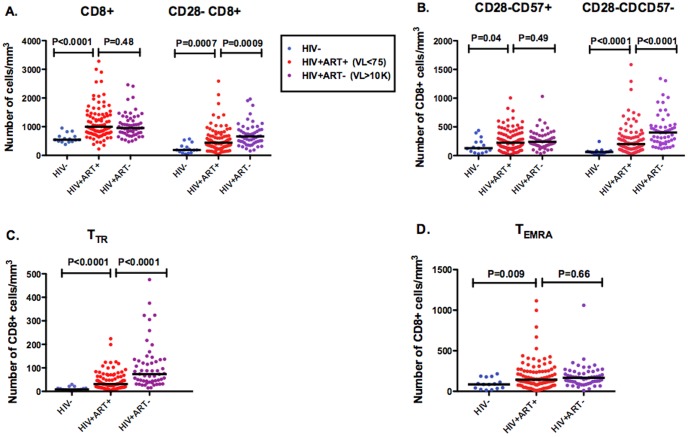
Effects of HIV and ART on the proportion of CD28-CD8+ T cells expressing CD57. The proportions of CD28- CD8+ T cells, CD28-CD57+ CD8+ T cells (**A**), of CD28-CD8+ T cells expressing CD57 (**B**), and of maturational subsets of CD28- CD8+ T cells: transitional memory, T_TR_, (CD28-CD27+CCR7-CD45RA-) and terminally differentiated, T_TEMRA_, (CD28-CD27-CCR7-CD45RA+) CD8+ T cells (**C**), were compared between HIV-uninfected (blue), ART-suppressed (red), and untreated viremic (purple) HIV-infected individuals. All comparisons were restricted to CMV-infected participants. Bars represent median values. The correlation between the proportion of CD28-CD8+ T cells expressing CD57 and the percent T_TR_ CD8+ T cells was also assessed in HIV-infected ART-suppressed participants (**D**).

To assess differences between HIV-uninfected and HIV-infected individuals, we restricted all analyses to CMV-infected participants since nearly all HIV-infected individuals in our cohorts are CMV co-infected. HIV-infected participants were mostly men and slightly older than the HIV-uninfected participants (Table 1). While the 55 untreated HIV-infected participants had a higher median percent CD28- CD8+ T cells than HIV-uninfected controls (69% vs. 38%, P<0.0001), they had a much lower median proportion of CD28-CD8+ T cells expressing CD57(39% vs. 64%, P<0.0001, Figure 2A–B). These competing effects resulted in similar percentages of CD28-CD57+ CD8+ T cells between HIV-infected and uninfected participants. While the 96 ART-suppressed participants had a higher median proportion of CD28-CD8+ T cells expressing CD57 than untreated participants (52% vs. 39%, P<0.0001), they had lower levels than HIV-uninfected controls (P = 0.001), despite a median of 4 years of ART-mediated viral suppression (Figure 2B). Untreated and ART-suppressed HIV-infected participants also had abnormally low proportions of CD57+ cells within each CD28- CD8+ T cell maturational subset compared to HIV negative controls (P≤0.03, Figure S2). The abnormally low proportion of CD28-CD8+ T cells expressing CD57 in untreated and ART-suppressed participants appeared to be primarily explained by an expansion of CD28-CD57- CD8+ T cells rather than a decline in CD28-CD57+ CD8+ T cell counts (Figure 3B). Thus, while CMV and aging appear to expand CD28-CD57+ CD8+ T cell populations, HIV appears to preferentially expand CD28-CD57- CD8+ populations, an effect which fails to completely normalize with suppressive ART.

Consistent with previously published work [12], untreated HIV-infected participants also had a higher median percent and count of transitional memory, T_TR_, (CD28-CCR7-CD27+CD45RA-) CD8+ T cells than HIV-uninfected participants (7.3% vs.1.8%, P<0.0001; 74 vs. 8 cells/mm^3^, P<0.0001, Figure 2C and 3C). While ART-suppressed participants had somewhat lower percent T_TR_ CD8+ T cells than untreated participants, they continued to have higher median levels than HIV-uninfected controls (2.9% vs. 1.8%, P = 0.0005). Among ART-suppressed participants, lower proportions of CD28-CD8+ T cells expressing CD57 were also associated with higher %T_TR_ CD8+ T cells (R = −0.25, P = 0.01, Figure 2D).

### Longitudinal ART-mediated changes in CD8+ T cell differentiation and CD57 expression

**Figure 4:**
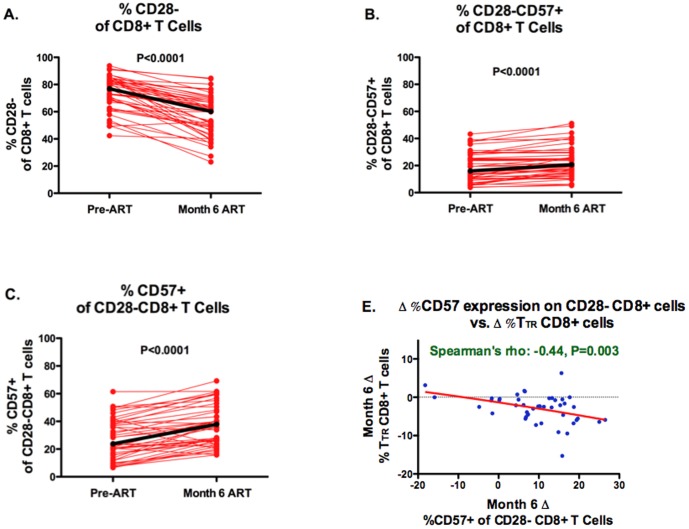
Impact of initial ART-mediated viral suppression on CD8+ T cell CD57 expression and percent transitional cells. Changes in the percent of CD28- CD8+ T cells (**A**), CD28-CD57+ CD8+ T cells (**B**), CD28-CD8+ T cells expressing CD57 (**C**), and CD28- transitional memory, T_TR_, (CD27+CCR7-CD45RA-) CD8+ T cells (**D**) are plotted over the first 6 months of ART-mediated viral suppression is plotted for 45 chronically HIV-infected Ugandans initiating their first ART regimen. Individual trajectories are shown in red and median trajectories with heavy black lines. The correlation between the ART-mediated change in the proportion of CD28-CD8+ T cells expressing CD57 and the ART-mediated change in %T_TR_ CD8+ T cells was also assessed (**E**).

To characterize longitudinal changes in CD8+ T cell differentiation and CD57 expression during early ART, we assessed 45 ART-naïve HIV-infected adult Ugandans before and after 6 months of suppressive ART in the UARTO cohort. Participants were mostly female and young, and had relatively low pre-ART CD4+ counts and high pre-ART HIV RNA levels (Table 2). While the median percent CD28- CD8+ T cells decreased during early ART (77% to 60%, P<0.0001), the percent CD28-CD57+ CD8+ T cells increased (16% to 21%, P<0.0001), as did the proportion of CD28-CD8+ T cells expressing CD57 (24% to 38%, P<0.0001, Figures 4A–C). The percent of CD8+ T_TR_ cells also declined during early ART (Figure 4D) and those experiencing the greatest declines in percent CD8+ T_TR_ also tended to experience the greatest increase in the proportion of CD28-CD8+ T cells expressing CD57 (rho: −0.44, P = 0.003, Figure 4E).

**Table 2:** Characteristics of HIV-infected Ugandans starting ART.

Characteristic	Median (IQR) (n = 45)
**Age, years**	36 (31–40)
**Male gender, No., (%)**	15 (33%)
**Pre-ART nadir CD4+ count, cells/mm^3^**	176 (124–236)
**Pre-ART plasma HIV RNA level, log_10_copies/mL**	5.1 (4.7–5.8)
**Month 6 CD4+ count, cells/mm^3^**	269 (211–388)

**Figure 5:**
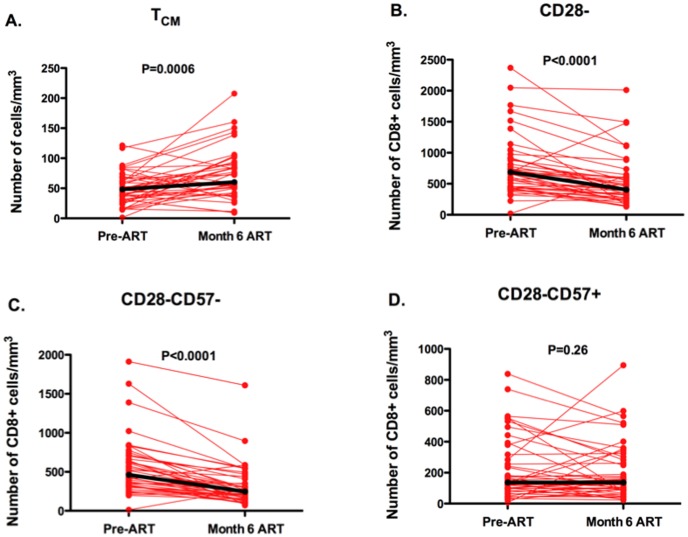
Impact of initial ART-mediated viral suppression on CD8+ T cell subset counts. Changes in central memory, T_CM_, (CD28+CD27+CCR7+CD45RA-) (**A**), CD28- (**B**), CD28-CD57- (**C**), and CD28-CD57+ CD8+ T cell counts (**D**) are plotted over the first six months of ART-mediated viral suppression for 45 chronically HIV-infected Ugandans initiating their first ART regimen. Individual trajectories are shown in red and median trajectories with heavy black lines.

The increase in the proportion of CD28-CD8+ T cells expressing CD57 during early ART appeared to be driven by a decline in CD28-CD57- CD8+ T cell counts (median 463 to 246 cells/mm^3^, P<0.0001, Figure 5C), without evidence for a change in CD28-CD57+ CD8+ T cell counts (median 137 to 137 cells, P = 0.26, Figure 5D). While median central memory CD8+ T cell counts actually increased during 6 months of ART (48 to 60 cells/mm^3^, P = 0.0006, Figure 5A), all maturational subsets of CD28- CD8+ T cells declined: T_TR_ (52 to 24 cells/mm^3^, P<0.0001), T_EM_, effector memory (CD28-CD27-CCR7-CD45RA-, 129 to 69 cells/mm^3^, P<0.0001), and T_EMRA_ (126 to 103 cells/mm^3^, P = 0.05) cells, with the smallest declines in the most terminally differentiated T_EMRA_ cells (Figure S3).

## Discussion

While CD57 expression on CD8+ T cells has long been associated with CMV infection, immunosenescence, and mortality risk in elderly HIV-uninfected populations [11], [13], [14], the impact of HIV on this marker has been less well characterized. In the current study, we confirm that CMV infection and advancing age increase CD57 expression on CD28- CD8+ T cells even in a much younger HIV-uninfected population than has been previously described [11]. We also demonstrated that unlike CMV and aging, HIV causes an expansion of less well-differentiated transitional memory CD8+ T cells and CD28-CD57- CD8+ T cells (resulting in an abnormally low proportion of CD28-CD8+ T cells expressing CD57), which partially reverses, but fail to normalize during suppressive ART. Thus, the phenotypic abnormalities in CD8+ T cells associated with both untreated and treated HIV infection are actually quite distinct from those associated with CMV infection and aging.

Aging and CMV both increase the proportion of CD28-CD8+ T cells expressing CD57 [2], [15], and higher percent CD28-CD57+ CD8+ T cells predict mortality in the HIV-uninfected elderly population [11]. Many have hypothesized that the chronic inflammatory state of HIV infection would cause similar CD8+ T cell phenotypic defects [16], [17]. Although HIV infection leads to an expansion of CD28- CD8+ T cells similar to that observed with CMV and aging, HIV results in an abnormally low proportion of these cells that express CD57. This phenomenon may be the reason why prior studies failed to observe decreases in percent CD28-CD57+ CD8+ T cells during ART [18], [19], since the percent CD28- CD8+ T cells declines while the proportion of these cells expressing CD57 increases during treatment. Indeed, the clinical relevance of the percent CD28-CD57+ CD8+ T cells is unclear in HIV infection. While a single study suggested that higher percent CD28-CD57+ CD8+ T cells were associated with greater atherosclerosis in HIV-infected women, it was unclear whether this association was independent of CD8+ T cell activation (or innate immune activation) [9]. The only two studies to date that have assessed the association between the percent CD28-CD57+ CD8+ T cells and mortality in treated HIV infection have tended to show decreased mortality in ART-suppressed individuals with higher frequencies of these cells [20], [21]. This raises the possibility that a decreased proportion of CD28-CD8+ T cells expressing CD57 in HIV-infected individuals might actually predict increased morbidity and mortality, a question we are addressing in a separate study [22].

**Figure 6:**
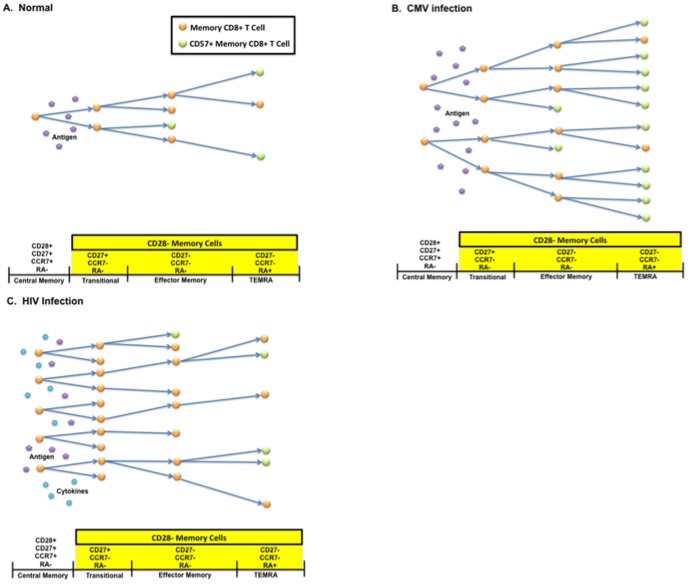
Schematic of CD8+ T cell differentiation and CD57 expression in HIV-uninfected and HIV-infected individuals. In HIV-uninfected and CMV-uninfected individuals, when a central memory CD8+ T cell sees its cognate antigen, it undergoes a burst of cellular proliferation and differentiation, and many - but not all - effector CD28-CD8+ T cells complete terminal differentiation and/or undergo several rounds of cell division (**A**). The cells that have undergone multiple rounds of proliferation and become terminally differentiated tend to express CD57. Asymptomatic CMV infection causes even greater proliferation and terminal differentiation of CD28-CD8+ T cells, resulting in multiple completed rounds of cell division and enrichment for T_EMRA_ cells that express CD57 (**B**). When CMV-seropositive individuals are infected with HIV, they experience an even greater expansion of CD28-CD8+ T cells, but a smaller proportion of these cells undergo successful proliferation and terminal differentiation, resulting in enrichment for less well-differentiated T_TR_ cells and lower CD57 expression on CD28- cells (**C**). CD57- memory CD8+ T cells are shown in orange, and cells expressing CD57 shown in green.

The physiologic significance of abnormally low proportions of CD28-CD8+ T cells expressing CD57 in HIV infection remains unclear. Much of the discussion on CD57+ CD8+ T cells in the aging literature has focused on the impaired ability of these cells to proliferate upon *in vitro* stimulation and their propensity to secrete inflammatory cytokines, properties that have been used to explain the observed association between high CD57 expression and mortality in the elderly population [23], [24], [25], [26], [27], [28], [29], [30], [31], [32], [33], [34], [35]. Nevertheless, high CD57 expression may also reflect a greater proliferative history of memory CD8+ T cells *in vivo*. Indeed, CD57+ CD8+ T cells at any given maturational stage have shorter telomere length and lower T cell receptor excision circle (TREC) content than CD57- CD8+ T cells from HIV-infected individuals [3]. Thus, abnormally low proportions of effector CD8+ T cells expressing CD57 in both untreated and treated HIV infection may reflect an impaired ability to successfully complete terminal differentiation and multiple rounds of cell division in response to antigens *in vivo*, which might result in a functional immune defect that is distinct from what is observed in CMV and aging. This concept is illustrated in our proposed model, whereby naïve and central memory CD8+ T cells normally give rise to multiple rounds of effector CD8+ T cell proliferation and terminal differentiation upon exposure to their cognate antigens (Figure 6A). The resultant CD57+ T_EMRA_ cells may be resistant to further proliferation *in vitro*, but are likely highly effective at killing infected cells [36]. This hypothesis is consistent with an early observation that higher percent CD57+ CD8+ T cells are associated with lower plasma HIV RNA level set-points in individuals with recent HIV infection [37]. Chronic antigenic stimulation due to CMV infection appears to accelerate cell proliferation and differentiation leading to enrichment for T_EMRA_ CD8+ T cells which have undergone several rounds of cell division and subsequently express CD57 (Figure 6B). We propose that in HIV infection, while inflammatory cytokines and antigens drive more naïve and central memory CD8+ T cells into cell cycle, the resultant effector CD28- CD8+ T cells undergo fewer rounds of proliferation and fail to terminally differentiate [22], leading to enrichment for less well-differentiated transitional memory cells, with perhaps poorer killing capacity (Figure 6C). Given recent data that CD57+ cells express chemokine receptors such as CX3CR1 that promote migration to non-lymphoid tissues [38], there is the possibility our observations may simply be due to the redistribution of CD28-CD8+ T cells that express CD57 into highly infected tissues. Therefore, our hypothesized model will need to be formally tested in subsequent *in vitro* and *in vivo* studies directly measuring T cell proliferation, cytotoxicity, and/or vaccine responsiveness and/or examining changes in tissues to determine whether the HIV-associated decrease in the proportion of CD28-CD8+ T cells expressing CD57 truly reflects a functional effector T cell defect. Non-isotopic labeling studies may also be help clarify the degree to which the expansion of CD28-CD57- CD8+ T cells observed in HIV infection is driven by increased proliferation versus a failure of these cells to die or terminally differentiate. Finally, the cross-sectional and longitudinal cohorts are from very different populations with potentially different co-pathogens that could have affected the percentage of CD28-CD8+ T cells expressing of CD57. However, the inferences were very similar between these cohorts, suggesting that the effect of HIV on this phenotype is likely to be independent of these demographic and environmental differences. We also observed similar results in another U.S.-based longitudinal cohort of individuals [22]. Lastly, it is important to emphasize that both cohorts in the current study had very advanced HIV infection at the time of ART initiation, so it remains unclear whether these results would be generalizable to HIV-infected individuals initiating ART at earlier stages of infection.

In summary, we found that the persistent phenotypic CD8+ T cell defects of treated HIV infection are distinct from those observed with CMV infection or aging-associated immunosenescence. While HIV leads to an expansion of CD28- CD8+ T cells, fewer of these cells may be able to successfully complete T cell maturation and proliferation, leading to an overall decrease in the proportion of these cells that express CD57. By examining the proportion of CD28- CD8+ T cells that express CD57 (rather than the proportion of the entire CD8+ T cell population expressing CD57 as has been assessed in prior studies [3], [4], [9]), our study provides insights into a unique CD8+ T abnormality that appears to be different from aging-associated immunosenescence. Future studies will need to clarify the prognostic importance of these abnormalities on clinical outcomes as well as the impact of these abnormalities on CD8+ T cell function in HIV-infected individuals.

## Supporting Information

Figure S1 **CD57 expression on CD28+ and CD28- CD8 + T cells in HIV-uninfected individuals.** A flow cytometry plot from a representative HIV-uninfected CMV-seropositive individual assessing CD28 and CD57 expression on CD8+ T cells (gated on total CD3+CD8+ T cells, CD57 gate set with FMO control) is shown (**A**). The percent of each population relative to the total CD8+ T cell population is depicted in blue font and the proportion of CD28+ and CD28- cells expressing CD57 is depicted in green font. The proportion of CD28+ (orange) and CD28- (purple) CD8+ T cells expressing CD57 was also compared among all HIV-uninfected individuals (**B**), demonstrating that CD57 expression is primarily observed among CD28- CD8+ T cells.
(TIFF)

Figure S2 **Effects of HIV and ART on the proportion of CD28-CD8+ T cells expressing CD57 by maturational subset.** The proportion of transitional memory, T_TR_, (CD27+CCR7-CD45RA-) (**A**), effector memory, T_EM_, (CD27-CCR7-CD45RA-) (**B**), and terminally differentiated, T_TEMRA_, (CD27-CCR7-CD45RA+) (**C**) CD28- CD8+ T cells that express CD57 were compared between HIV-uninfected individuals (blue), HIV+ ART-suppressed (red), and HIV+ untreated viremic (purple) individuals. Bars represent median values. All comparisons were restricted to CMV-positive individuals.
(TIFF)

Figure S3 **Impact of ART-mediated viral suppression on cell counts of CD8+ T cell maturational subsets.** Changes in the cell counts of central memory, T_CM_, (CD28+CD27+CCR7+CD45RA-) (**A**), CD28- transitional memory, T_TR_, (CD28-CD27+CCR7-CD45RA-) (**B**), effector memory, T_EM_ (CD28-CD27-CCR7-CD45RA-) (**C**), and terminally differentiated, T_EMRA_ (CD28-CD27-CCR7-CD45RA+) CD8+ T cells (**D**) are plotted over the first six months of ART-mediated viral suppression for 45 HIV-infected Ugandans initiating their first ART regimen. Individual trajectories are shown in red and median trajectories with heavy black lines.
(TIFF)

## References

[1] Deeks SG (2011) HIV infection, inflammation, immunosenescence, and aging. Annu Rev Med 62: 141–155. 21090961 10.1146/annurev-med-042909-093756 PMC3759035

[2] Dock JN, Effros RB (2011) Role of CD8 T Cell Replicative Senescence in Human Aging and in HIV-mediated Immunosenescence. Aging and disease 2: 382–397. 22308228 PMC3269814

[3] Brenchley JM, Karandikar NJ, Betts MR, Ambrozak DR, Hill BJ, et al (2003) Expression of CD57 defines replicative senescence and antigen-induced apoptotic death of CD8+ T cells. Blood 101: 2711–2720. 12433688 10.1182/blood-2002-07-2103

[4] Appay V, Fastenackels S, Katlama C, Ait-Mohand H, Schneider L, et al (2011) Old age and anti-cytomegalovirus immunity are associated with altered T-cell reconstitution in HIV-1-infected patients. Aids 25: 1813–1822. 21412126 10.1097/QAD.0b013e32834640e6

[5] Papagno L, Spina CA, Marchant A, Salio M, Rufer N, et al (2004) Immune Activation and CD8(+) T-Cell Differentiation towards Senescence in HIV-1 Infection. PLoS Biol 2: E20. 14966528 10.1371/journal.pbio.0020020 PMC340937

[6] Ladell K, Hellerstein MK, Cesar D, Busch R, Boban D, et al (2008) Central memory CD8+ T cells appear to have a shorter lifespan and reduced abundance as a function of HIV disease progression. Journal of immunology 180: 7907–7918. 10.4049/jimmunol.180.12.7907 PMC2562545 18523254

[7] Hatano H, Jain V, Hunt PW, Lee TH, Sinclair E, et al (2013) Cell-Based Measures of Viral Persistence Are Associated With Immune Activation and Programmed Cell Death Protein 1 (PD-1)-Expressing CD4+ T cells. The Journal of Infectious Diseases 208: 50–56. 23089590 10.1093/infdis/jis630 PMC3666131

[8] Jain V, Hartogensis W, Bacchetti P, Hunt PW, Hatano H, et al.. (2013) Antiretroviral therapy initiated within 6 months of HIV infection is associated with lower T-cell activation and smaller HIV reservoir size. Journal of Infectious Diseases In press. 10.1093/infdis/jit311 PMC3778965 23852127

[9] Kaplan RC, Sinclair E, Landay AL, Lurain N, Sharrett AR, et al (2011) T cell activation and senescence predict subclinical carotid artery disease in HIV-infected women. J Infect Dis 203: 452–463. 21220772 10.1093/infdis/jiq071 PMC3071219

[10] Naeger DM, Martin JN, Sinclair E, Hunt PW, Bangsberg DR, et al (2010) Cytomegalovirus-specific T cells persist at very high levels during long-term antiretroviral treatment of HIV disease. PLoS ONE 5: e8886. 20126452 10.1371/journal.pone.0008886 PMC2813282

[11] Wikby A, Mansson IA, Johansson B, Strindhall J, Nilsson SE (2008) The immune risk profile is associated with age and gender: findings from three Swedish population studies of individuals 20–100 years of age. Biogerontology 9: 299–308. 18369735 10.1007/s10522-008-9138-6

[12] Appay V, Dunbar PR, Callan M, Klenerman P, Gillespie GM, et al (2002) Memory CD8+ T cells vary in differentiation phenotype in different persistent virus infections. Nature medicine 8: 379–385. 10.1038/nm0402-379 11927944

[13] Trzonkowski P, Mysliwska J, Szmit E, Wieckiewicz J, Lukaszuk K, et al (2003) Association between cytomegalovirus infection, enhanced proinflammatory response and low level of anti-hemagglutinins during the anti-influenza vaccination–an impact of immunosenescence. Vaccine 21: 3826–3836. 12922116 10.1016/s0264-410x(03)00309-8

[14] Wang EC, Moss PA, Frodsham P, Lehner PJ, Bell JI, et al (1995) CD8highCD57+ T lymphocytes in normal, healthy individuals are oligoclonal and respond to human cytomegalovirus. Journal of immunology 155: 5046–5056. 7594513

[15] Wikby A, Johansson B, Olsson J, Lofgren S, Nilsson BO, et al (2002) Expansions of peripheral blood CD8 T-lymphocyte subpopulations and an association with cytomegalovirus seropositivity in the elderly: the Swedish NONA immune study. Experimental gerontology 37: 445–453. 11772532 10.1016/s0531-5565(01)00212-1

[16] Deeks SG, Verdin E, McCune JM (2012) Immunosenescence and HIV. Current opinion in immunology 24: 501–506. 22658763 10.1016/j.coi.2012.05.004

[17] Effros RB, Fletcher CV, Gebo K, Halter JB, Hazzard WR, et al (2008) Aging and infectious diseases: workshop on HIV infection and aging: what is known and future research directions. Clin Infect Dis 47: 542–553. 18627268 10.1086/590150 PMC3130308

[18] Plana M, Garcia F, Gallart T, Tortajada C, Soriano A, et al (2000) Immunological benefits of antiretroviral therapy in very early stages of asymptomatic chronic HIV-1 infection. AIDS 14: 1921–1933. 10997396 10.1097/00002030-200009080-00007

[19] Tortajada C, Garcia F, Plana M, Gallart T, Maleno MJ, et al (2000) Comparison of T-cell subsets' reconstitution after 12 months of highly active antiretroviral therapy initiated during early versus advanced states of HIV disease. Journal of acquired immune deficiency syndromes 25: 296–305. 11114829 10.1097/00042560-200012010-00002

[20] Hunt P, Rodriguez B, Shive C, Clagett B, Funderburg N, et al.. (2012) Gut Epithelial Barrier Dysfunction, Inflammation, and Coagulation Predict Higher Mortality during Treated HIV/AIDS. In the Program and Abstracts from the 19th Conference on Retroviruses and Opportunistic Infections, Seattle, WA, Abstract #278.

[21] Tenorio A, Zheng E, Bosch R, Deeks SG, Rodriguez B, et al.. (2013) Soluble Markers of Inflammation & Coagulation, but not T-Cell Activation, Predict Non-AIDS-Defining Events During Suppressive Antiretroviral Therapy; in the Program and Abstracts of the 20th Conference on Retroviruses and Opportunistic Infections, 2013, Atlanta, GA, Abstract #790.

[22] Lee SA, Sinclair E, Jain V, Huang Y, Epling L, et al.. (2014) Low Proportions of CD28- CD8+ T cells Expressing CD57 Can Be Reversed by Early ART Initiation and Predict Mortality in Treated HIV. Infection J Infect Dis: in press. 10.1093/infdis/jiu109 PMC4110459 24585893

[23] Ferguson FG, Wikby A, Maxson P, Olsson J, Johansson B (1995) Immune parameters in a longitudinal study of a very old population of Swedish people: a comparison between survivors and nonsurvivors. J Gerontol A Biol Sci Med Sci 50: B378–382. 7583794 10.1093/gerona/50a.6.b378

[24] Wikby A, Ferguson F, Forsey R, Thompson J, Strindhall J, et al (2005) An immune risk phenotype, cognitive impairment, and survival in very late life: impact of allostatic load in Swedish octogenarian and nonagenarian humans. J Gerontol A Biol Sci Med Sci 60: 556–565. 15972602 10.1093/gerona/60.5.556

[25] Olsson J, Wikby A, Johansson B, Lofgren S, Nilsson BO, et al (2000) Age-related change in peripheral blood T-lymphocyte subpopulations and cytomegalovirus infection in the very old: the Swedish longitudinal OCTO immune study. Mech Ageing Dev 121: 187–201. 11164473 10.1016/s0047-6374(00)00210-4

[26] Strindhall J, Nilsson BO, Lofgren S, Ernerudh J, Pawelec G, et al (2007) No Immune Risk Profile among individuals who reach 100 years of age: findings from the Swedish NONA immune longitudinal study. Exp Gerontol 42: 753–761. 17606347 10.1016/j.exger.2007.05.001

[27] Merino J, Martinez-Gonzalez MA, Rubio M, Inoges S, Sanchez-Ibarrola A, et al (1998) Progressive decrease of CD8high+ CD28+ CD57- cells with ageing. Clin Exp Immunol 112: 48–51. 9566789 10.1046/j.1365-2249.1998.00551.x PMC1904936

[28] Wikby A, Johansson B, Olsson J, Lofgren S, Nilsson BO, et al (2002) Expansions of peripheral blood CD8 T-lymphocyte subpopulations and an association with cytomegalovirus seropositivity in the elderly: the Swedish NONA immune study. Exp Gerontol 37: 445–453. 11772532 10.1016/s0531-5565(01)00212-1

[29] Koch S, Larbi A, Ozcelik D, Solana R, Gouttefangeas C, et al (2007) Cytomegalovirus infection: a driving force in human T cell immunosenescence. Ann N Y Acad Sci 1114: 23–35. 17986574 10.1196/annals.1396.043

[30] Vasto S, Colonna-Romano G, Larbi A, Wikby A, Caruso C, et al (2007) Role of persistent CMV infection in configuring T cell immunity in the elderly. Immun Ageing 4: 2. 17376222 10.1186/1742-4933-4-2 PMC1831794

[31] Hadrup SR, Strindhall J, Kollgaard T, Seremet T, Johansson B, et al (2006) Longitudinal studies of clonally expanded CD8 T cells reveal a repertoire shrinkage predicting mortality and an increased number of dysfunctional cytomegalovirus-specific T cells in the very elderly. J Immunol 176: 2645–2653. 16456027 10.4049/jimmunol.176.4.2645

[32] Ouyang Q, Wagner WM, Zheng W, Wikby A, Remarque EJ, et al (2004) Dysfunctional CMV-specific CD8(+) T cells accumulate in the elderly. Exp Gerontol 39: 607–613. 15050296 10.1016/j.exger.2003.11.016

[33] Ouyang Q, Wagner WM, Wikby A, Walter S, Aubert G, et al (2003) Large numbers of dysfunctional CD8+ T lymphocytes bearing receptors for a single dominant CMV epitope in the very old. J Clin Immunol 23: 247–257. 12959217 10.1023/a:1024580531705

[34] Almanzar G, Schwaiger S, Jenewein B, Keller M, Herndler-Brandstetter D, et al (2005) Long-term cytomegalovirus infection leads to significant changes in the composition of the CD8+ T-cell repertoire, which may be the basis for an imbalance in the cytokine production profile in elderly persons. J Virol 79: 3675–3683. 15731261 10.1128/JVI.79.6.3675-3683.2005 PMC1075718

[35] Khan N, Shariff N, Cobbold M, Bruton R, Ainsworth JA, et al (2002) Cytomegalovirus seropositivity drives the CD8 T cell repertoire toward greater clonality in healthy elderly individuals. J Immunol 169: 1984–1992. 12165524 10.4049/jimmunol.169.4.1984

[36] Hersperger AR, Martin JN, Shin LY, Sheth PM, Kovacs CM, et al (2011) Increased HIV-specific CD8+ T-cell cytotoxic potential in HIV elite controllers is associated with T-bet expression. Blood 117: 3799–3808. 21289310 10.1182/blood-2010-12-322727 PMC3083297

[37] Lieberman J, Trimble LA, Friedman RS, Lisziewicz J, Lori F, et al (1999) Expansion of CD57 and CD62L-CD45RA+ CD8 T lymphocytes correlates with reduced viral plasma RNA after primary HIV infection. AIDS 13: 891–899. 10371169 10.1097/00002030-199905280-00004

[38] Combadiere B, Faure S, Autran B, Debre P, Combadiere C (2003) The chemokine receptor CX3CR1 controls homing and anti-viral potencies of CD8 effector-memory T lymphocytes in HIV-infected patients. AIDS 17: 1279–1290. 12799549 10.1097/00002030-200306130-00002

