# Supplementary figures and images for: Impact of HIV on CD8+ T Cell CD57 Expression Is Distinct from That of CMV and Aging
Source: PLoS One. 2014 Feb 27;9(2):e89444. doi: 10.1371/journal.pone.0089444 (PMC3937334; doi:10.1371/journal.pone.0089444)

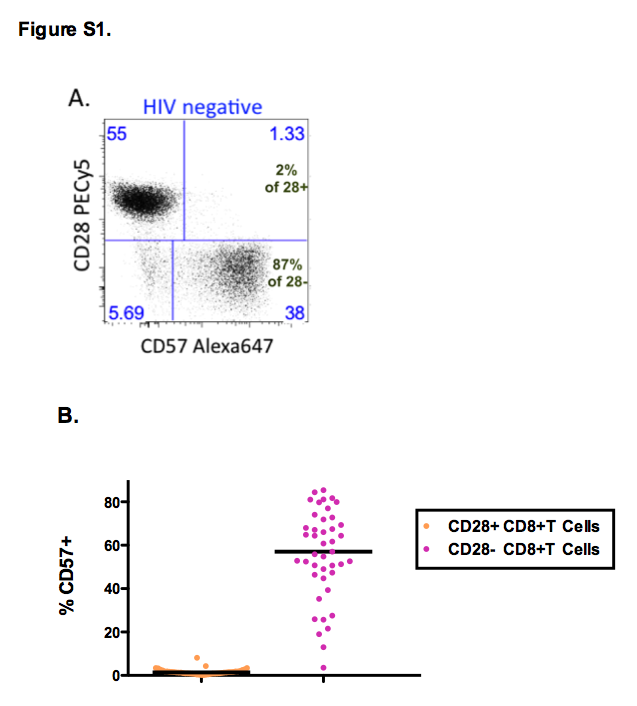

Supplement: Figure S1 — CD57 expression on CD28+ and CD28- CD8 + T cells in HIV-uninfected individuals. A flow cytometry plot from a representative HIV-uninfected CMV-seropositive individual assessing CD28 and CD57 expression on CD8+ T cells (gated on total CD3+CD8+ T cells, CD57 gate set with FMO control) is shown (A). The percent of each population relative to the total CD8+ T cell population is depicted in blue font and the proportion of CD28+ and CD28- cells expressing CD57 is depicted in green font. The proportion of CD28+ (orange) and CD28- (purple) CD8+ T cells expressing CD57 was also compared among all HIV-uninfected individuals (B), demonstrating that CD57 expression is primarily observed among CD28- CD8+ T cells. (TIFF) [file pone.0089444.s001.tiff]

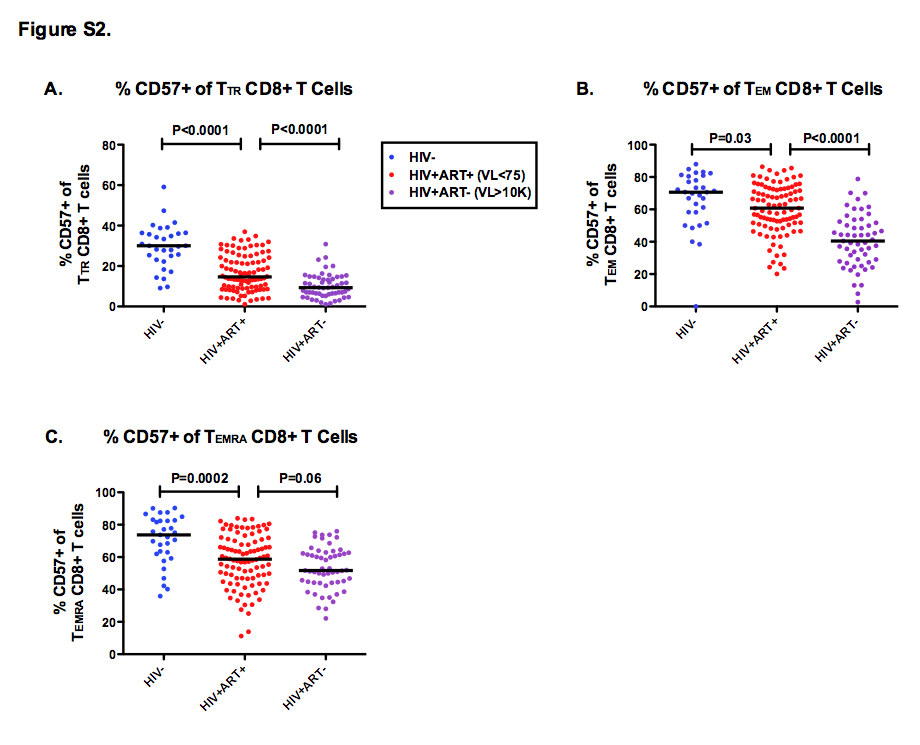

Supplement: Figure S2 — Effects of HIV and ART on the proportion of CD28-CD8+ T cells expressing CD57 by maturational subset. The proportion of transitional memory, TTR, (CD27+CCR7-CD45RA-) (A), effector memory, TEM, (CD27-CCR7-CD45RA-) (B), and terminally differentiated, TTEMRA, (CD27-CCR7-CD45RA+) (C) CD28- CD8+ T cells that express CD57 were compared between HIV-uninfected individuals (blue), HIV+ ART-suppressed (red), and HIV+ untreated viremic (purple) individuals. Bars represent median values. All comparisons were restricted to CMV-positive individuals. (TIFF) [file pone.0089444.s002.tiff]

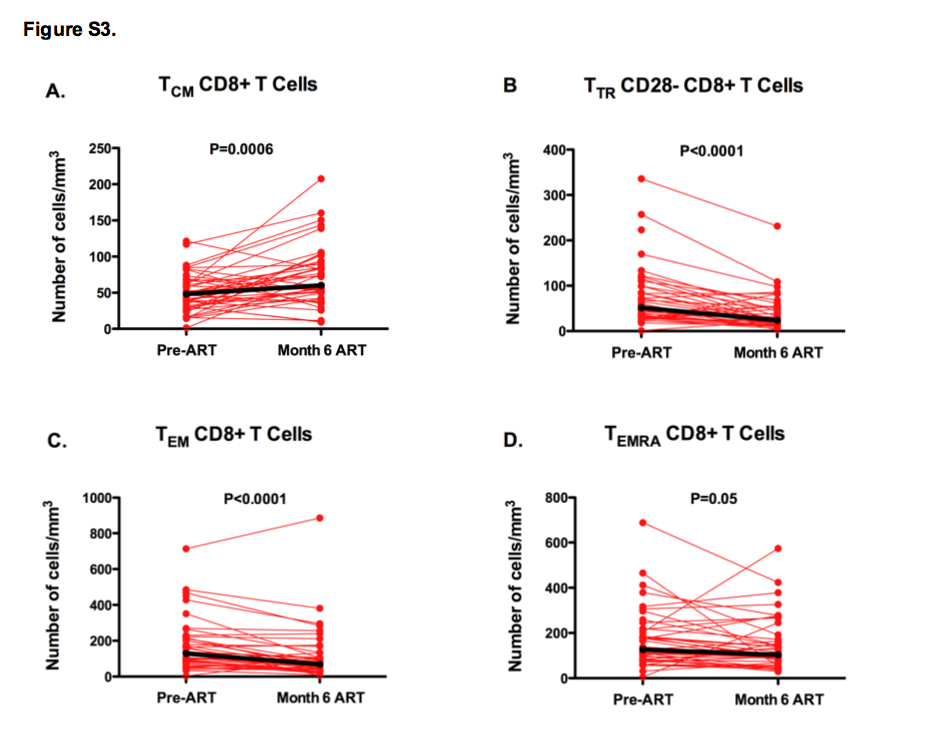

Supplement: Figure S3 — Impact of ART-mediated viral suppression on cell counts of CD8+ T cell maturational subsets. Changes in the cell counts of central memory, TCM, (CD28+CD27+CCR7+CD45RA-) (A), CD28- transitional memory, TTR, (CD28-CD27+CCR7-CD45RA-) (B), effector memory, TEM (CD28-CD27-CCR7-CD45RA-) (C), and terminally differentiated, TEMRA (CD28-CD27-CCR7-CD45RA+) CD8+ T cells (D) are plotted over the first six months of ART-mediated viral suppression for 45 HIV-infected Ugandans initiating their first ART regimen. Individual trajectories are shown in red and median trajectories with heavy black lines. (TIFF) [file pone.0089444.s003.tiff]
